# *Curcuma longa* Is Able to Induce Apoptotic Cell Death of Pterygium-Derived Human Keratinocytes

**DOI:** 10.1155/2017/2956597

**Published:** 2017-12-17

**Authors:** Silvia Sancilio, Silvio Di Staso, Stefano Sebastiani, Lucia Centurione, Nick Di Girolamo, Marco Ciancaglini, Roberta Di Pietro

**Affiliations:** ^1^Department of Pharmacy, G. d'Annunzio University of Chieti-Pescara, Via dei Vestini 31, 66100 Chieti, Italy; ^2^Ophthalmic Clinic, Department of Life, Health and Environmental Sciences, L'Aquila University, Piazzale S. Tommasi 1, 67100 L'Aquila, Italy; ^3^Sebastiani Pharmacy, Via Italia 70, 65010 Spoltore, Italy; ^4^Department of Medicine and Ageing Science, School of Medicine and Health Sciences, G. d'Annunzio University of Chieti-Pescara, Via dei Vestini 31, 66100 Chieti, Italy; ^5^Inflammatory Diseases Research Unit, School of Medical Sciences, The University of New South Wales, Sydney, NSW 2052, Australia

## Abstract

Pterygium is a relatively common eye disease that can display an aggressive clinical behaviour. To evaluate the* in vitro* effects of* Curcuma longa* on human pterygium-derived keratinocytes, specimens of pterygium from 20 patients undergoing pterygium surgical excision were collected. Pterygium explants were put into culture and derived keratinocytes were treated with an alcoholic extract of 1.3%* Curcuma longa* in 0.001% Benzalkonium Chloride for 3, 6, and 24 h. Cultured cells were examined for CAM5.2 (anti-cytokeratin antibody) and CD140 (anti-fibroblast transmembrane glycoprotein antibody) expression between 3th and 16th passage to assess cell homogeneity. TUNEL technique and Annexin-V/PI staining in flow cytometry were used to detect keratinocyte apoptosis. We showed that* Curcuma longa *exerts a proapoptotic effect on pterygium-derived keratinocytes already after 3 h treatment. Moreover, after 24 h treatment,* Curcuma longa *induces a significant increase in TUNEL as well as Annexin-V/PI positive cells in comparison to untreated samples. Our study confirms previous observations highlighting the expression, in pterygium keratinocytes, of nuclear VEGF and gives evidence for the first time to the expression of nuclear and cytoplasmic VEGF-R1. All in all, these findings suggest that* Curcuma longa* could have some therapeutic potential in the treatment and prevention of human pterygium.

## 1. Introduction

Ocular surface epithelium is composed of corneal, conjunctival, and limbal epithelial layers [1]. Epithelia covering the corneal and the conjunctival surfaces are both stratified and nonkeratinized but only the conjunctival epithelium contains goblet cells involved in a gel-forming mucina secretion [2, 3]. Functionally, corneal and conjunctival epithelium play similar roles since both are able to be wettable so as to maintain comfort, while providing smooth optical surface [4], and to protect the underlying tissues from infection [2]. Limbal epithelium has a key role in the maintenance of corneal transparency by means of a population of limbal epithelial stem cells. Depletion of stratospheric ozone has increased the flux of ultraviolet-B (UV-B) radiation at the surface of the earth increasing the rate of UV-induced eye damage [5]. An ocular surface disease attributed to chronic UV-B exposure and to lesions of the limbal epithelium is pterygium [6]. Pterygium has been defined by Duke-Elder as a triangular shaped degenerative and hyperplastic process, occurring medially and laterally in the rhyme eyelid, in which the bulbar conjunctiva encroaches on the cornea [7]. Pterygium is a very common fibrovascular lesion widespread in the so called “pterygium zone” [8]. This is an area defined by a geographical latitude of 40° north and south of the equator. Here, a prevalence rate of up to 22% in the population has been reported. Outside this area prevalence rates usually do not overcome 2% of the general population and the disease affects mainly patients with an increased exposure to sunlight or involved in outdoor activities [9]. Although previous studies have highlighted the involvement of genetic factors in the pathogenesis of pterygium [10], the etiology of pterygium still remains unclear [8]. Current management strategies for pterygium imply surgical excision [11] that is a complex and invasive procedure that often results in the recurrence of a lesion more clinically aggressive than the original one [9]. In addition, surgery can lead to further clinical manifestations such as symblepharon, corneal clouding, corneal or scleral dellen [9, 12], keloid formation [13], and scleral necrosis [14].

*Curcuma longa* is a plant belonging to the family of Zingiberaceae. The rhizomes of this plant are the source of turmeric, which has been used for centuries all over the world in cooking, cosmetics, and medical treatments [15]. The curcuminoids present in the rhizome, which are responsible for the yellow colour of turmeric, consist of a mixture of curcumin (also known as diferuloylmethane, Natural Yellow, and E100), demethoxycurcumin, and bisdemethoxycurcumin [16]. Curcumin makes up ~90% of the curcuminoid content. Due to its chemical structure, curcumin is much less soluble in water at acid and neutral pH but soluble in methanol, ethanol, dimethyl sulfoxide (DMSO), and acetone [17]. Its traditional uses as a strong therapeutic or preventive agent in several human diseases such as diabetes, inflammation, atherosclerosis, and cancer [18] is due to its beneficial properties including anti-inflammatory, antioxidant, antineoplastic, pro- and antiapoptotic, antiangiogenic, cytotoxic, immune-modulatory, and antimicrobial effects* via* the modulation of various targets (grow factors, enzymes, and genes) [19].

Based on available literature and with the aim to identify an alternative strategy to currently available surgical procedures, in this study we investigated the effects of* in vitro* treatment with* Curcuma longa* of keratinocytes derived from explants of human pterygium.

## 2. Materials and Methods

### 2.1. Experimental Design

Human pterygium specimens were obtained from 20 patients with primary pterygium (average age 68.2 ± 9.7 years) undergoing routine pterygiectomy. All the patients affected with pterygia displayed inflammatory signs of the ocular surface such as chemosis and redness of the conjunctiva. Normal conjunctival tissue specimens were obtained from 3 patients (average age 71.2 ± 8.3 years) undergoing cataract surgery. All patients were treated at the SS. Annunziata Hospital in Chieti, Italy. Signed informed consent was obtained from the donors according to Italian legislation and to the code of Ethical Principles for Medical Research involving Human Subjects of the World Medical Association (Declaration of Helsinki). After surgical excision, the clinical specimens were properly treated or preserved for immunohistochemistry or primary cell culture.

### 2.2. Primary Culture and Subculture of Pterygium-Derived Keratinocytes

Pterygium specimens were placed in a Petri dish and washed with 1x Dulbecco's Phosphate-Buffered Saline (PBS). Samples were cut into several 1-2 mm^2^ pieces under sterile conditions and placed into six-well plates at 37°C in an atmosphere of 5% carbon dioxide in air. Explants were allowed to attach to the plastic dish for at least 8 h in a drop of complete medium selective for human keratinocytes survival and consisting of Eagle's MEM (GIBCO by Invitrogen, Carlsbad, CA, USA) supplemented with 10% foetal bovine serum (FBS; Hyclone, Logan, UT, USA), 100 U/ml penicillin, 100 *μ*g/ml streptomycin, and 2.5 *μ*g/ml fungizone (Sigma-Aldrich, St. Louis, MO, USA). Cell migration from explants was observed within 3–5 days. Cells were used for experiments between the 4th and 7th passage and only for characterization up to the 16th passage. Pterygium-derived keratinocytes were grown on 6-well culture plates and when cells reached confluence, culture medium was replaced and, where required, treated with an alcoholic extract of 1.3%* Curcuma longa* in 0.001% Benzalkonium Chloride (BCA) (Sigma-Aldrich), a well known preservative commonly added to several eye drops. Control cultures were left untreated or treated with the vehicle alone (BCA) for the same time intervals. After 3, 6, and 24 h observations were carried out under a phase contrast light microscope (LEICA, Wetzlar, Germany) equipped with a CoolSNAP video camera for acquiring computerized images (Photometrics, Tucson, AZ).

### 2.3. Histochemical Analysis

Explants of human pterygium were fixed with 10% (vol/vol) phosphate-buffered formalin, dehydrated in a series of graded increases in alcohol concentrations, embedded in paraffin and cut at the microtome (Leica, RM 2265, Germany). Sections were routinely stained with Haematoxylin-Eosin staining solution and mounted in Bio Mount (Bio-Optica, Italy). For periodic acid–Schiff (PAS) staining, samples were fixed with 4% paraformaldehyde, washed with PBS, oxidized in 1% periodic acid for 5 min, and rinsed in several changes of deionized water. The cells were then incubated with Schiff reagent (containing basic fuchsine, potassium metabisulfite, and hydrochloric acid) for 15 min and washed with tap water for 5 min, and their nuclei were counterstained with Mayer's Haematoxylin for 1 min. All the samples were observed under a ZEISS Axioskop 40 (Carl Zeiss, Gottingen, Germany) light microscope equipped with a CoolSNAP video camera (Photometrics, Tucson, AZ, USA) for acquiring digital images.

### 2.4. Characterization Studies

Cultured cells were examined for CAM 5.2 (anti-cytokeratin antibody) and CD140 (anti-fibroblast transmembrane glycoprotein antibody) expression between 3th and 16th passages. Cells were fixed for 10 min with a 3% paraformaldehyde solution at room temperature in 1x Dulbecco's PBS pH 7.6 supplemented with 2% sucrose. Then, cell membranes were permeabilized for 5 min at room temperature with a pH 7.6 solution containing 0.5% Triton X-100/20 mM HEPES, 300 *μ*M sucrose, 50 mM NaCl, and 3 mM MgCl_2_. After membrane permeabilization, cells were incubated with 10% BSA in 1x Dulbecco's PBS for 30 min at room temperature, followed by a 45 min incubation at room temperature with FITC-conjugated CAM 5.2 at the final concentration of 5 *μ*g/ml (in 1% BSA/PBS) and PE-conjugated CD140 antibody at the final concentration of 5 *μ*g/ml (in 1% BSA/PBS) (all purchased from BD, San Jose, CA, USA). All the slides were mounted with Slowfade (Molecular Probes, Eugene OR) and observed with a ZEISS Axioskop 40 light microscope equipped with a CoolSNAP video camera (Photometrics) for acquiring digital images.

### 2.5. Immunofluorescent Staining of DNA Strand Breaks (TUNEL)

The TUNEL assay detects single or double DNA strand breaks with the use of labelled nucleotides, polymerized to free 3′-hydroxyl termini in a reaction catalysed by Terminal deoxynucleotidyl Transferase (TdT). Cells were grown in a complete medium in 24-well plates and treated with 1.3%* Curcuma longa* in 0.001% BCA up to 24 h. Samples were fixed with 4% paraformaldehyde, firstly washed, and then incubated in a permeabilizing solution (0.1% Triton X-100, 0.1% sodium citrate) for 2 min on ice. Deoxyribonucleic acid strand breaks were identified with the use of an “in situ cell death detection kit” (Boehringer Mannheim, Mannheim, Germany) according to the manufacturer's instructions as elsewhere reported [20].

### 2.6. Annexin-V/PI Detection of Apoptotic and Necrotic Cells in Flow Cytometry

In order to assess apoptosis, a commercial Annexin-V-FITC/PI kit (Bender Med System, Vienna, Austria) was used according to the manufacturer's instructions as elsewhere reported [21]. Analyses were performed using a Coulter FC500 flow cytometer with the FL1 and the FL3 in a log mode with the use of the CXP Software (Beckman Coulter, FL, USA). For each sample, 10.000–20.000 events were collected. Viable cells were Annexin-V^neg^/PI^neg^ (unlabelled), early apoptotic cells were Annexin-V^pos^/PI^neg^, and late apoptotic cells were Annexin-V^pos^/PI^pos^, whereas necrotic cells were Annexin-V^neg^/PI^pos^.

### 2.7. Immunofluorescent Staining of VEGF and VEGF-R1

To evaluate the expression of vascular endothelial growth factor (VEGF) and its receptor (VEGF-R1) adherent cells were fixed, permeabilized, and blocked as mentioned above. Samples were then incubated with anti-human VEGF and VEGF-R1 (Becton Dickinson Biosciences, San Jose, California, USA) (working dilution, 1 : 500) followed by FITC-conjugated IgG and TRITC-conjugated IgG (working dilution, 1 : 100) (Jackson ImmunoResearch, West Grove, PA, USA), respectively, as secondary antibodies. Cell nuclei were counterstained with DAPI (Vector Laboratories, Inc., Burlingame, CA). All observations were performed under a ZEISS Axioskop 40 light microscope equipped with a CoolSNAP video camera (Photometrics) to acquire images to analyse with MetaMorph® 6.1 software (Universal Imaging Corp, Downingtown, PA, USA).

### 2.8. Statistics

Statistical analysis was performed using the analysis of variance (ANOVA) test. Results were expressed as means ± SD. Values of *p* < 0.05 were considered statistically significant.

## 3. Results

### 3.1. Histochemistry

**Figure 1:**
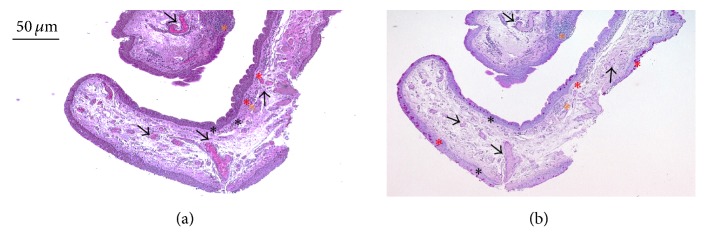
Typical histological features of pterygium. Two serial tissue sections of pterygium were stained (a) with Haematoxylin-Eosin and (b) with periodic acid–Schiff (PAS) staining solutions. Red asterisks show goblet cells grouped as intraepithelial glands, while black asterisks show goblet cells randomly placed within pterygium epithelium. Black arrows show a rich vascular network, orange asterisks show inflammatory infiltrates. Original magnification: 10x.

Surgical explants were subjected to histochemical analysis to assess morphological features. As shown in Figures 1(a) and 1(b), the pterygium epithelium, that centripetally invades the cornea, displays squamous metaplasia and goblet cell hyperplasia. As expected, the connective tissue underlying the multilayered epithelium is highly vascularized and full of inflammatory infiltrates (Figures 1(a) and 1(b)).

### 3.2. Primary Culture and Subculture of Pterygium-Derived Epithelial Cells

**Figure 2:**
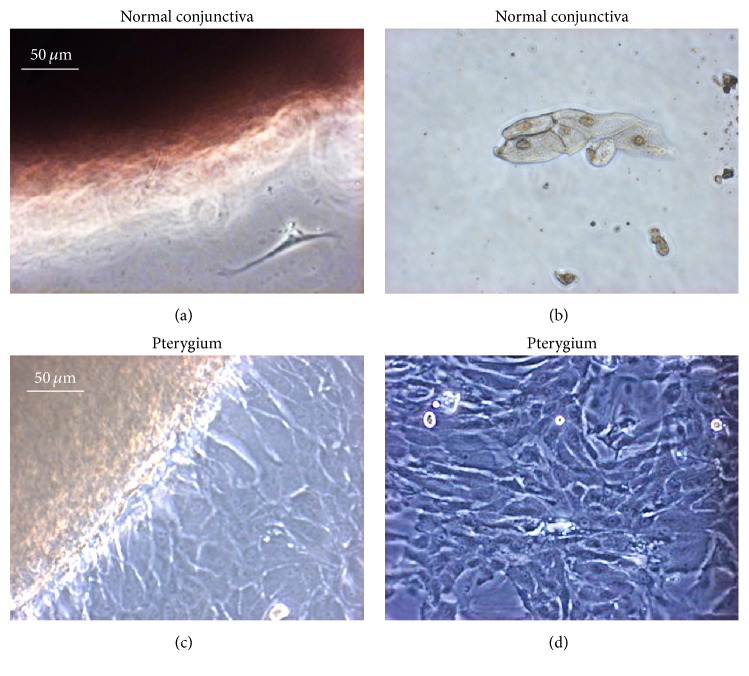
Phase contrast images of human keratinocytes derived from normal conjunctiva (a, b) and pterygium explants (c, d). On the left, images of the explants of normal conjunctiva (a) and pterygium (c) are shown. The cell overgrowth in the normal conjunctiva (a, b) was highly reduced compared with pterygium, where the overgrowth was really extensive. In both cases, cells displayed a typical polygonal morphology. Original magnification: 20x.

Cell outgrowth from explants was observed within 5–8 days in serum-supplemented media except for normal conjunctiva where only few cells were detectable near or far away from the explant (Figures 2(a) and 2(b)). Phase contrast microscopy revealed that primary outgrowths of cells exhibited a polygonal morphology typical of epithelial cells (Figures 2(b) and 2(c)). After 2 weeks in primary culture, cells were trypsinised and seeded onto uncoated tissue culture dishes in the presence of 10% FBS. Subcultured pterygium-derived epithelial cells appeared more irregular (Figure 2(d)) and grew rapidly in less well-organized monolayers up to 16 passages in culture.

### 3.3. Characterization Studies

**Figure 3:**
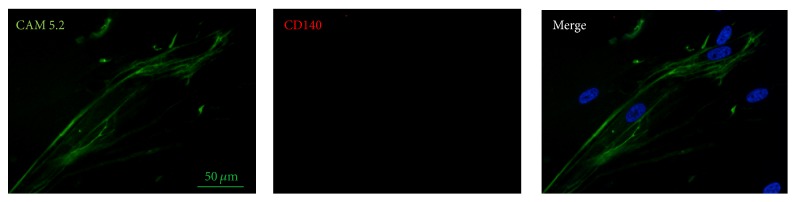
Immunofluorescence analysis of cultured human keratinocytes derived from pterygium explants labelled with anti-cytokeratin CAM 5.2 antibody to determine the purity of cultured cells (98 ± 6% positive cells) and with CD140 (no positive cells were detected) to verify fibroblastic contamination. Original magnification: 40x. The observations were carried out using a ZEISS Axioskop light microscope equipped with a Coolsnap video camera to acquire images analysed with the MetaMorph 6.1 Software (Universal Imaging Corp, Downingtown, PA). Images displayed are representative of 3 independent experiments.

To characterize pterygium-derived epithelial cells immune-histochemical detection of both keratinocyte (CAM 5.2) and fibroblast (CD140) markers was performed. The anti-cytokeratin (CAM 5.2) antibody is commonly used for qualitative identification of both normal and malignant cells of epithelial origin. To ascertain whether the primary cell population growing in culture did not contain contaminating fibroblasts we performed the immune-histochemical staining of cultured keratinocytes with anti-CD140 antibody. As shown in Figure 3 primary pterygium-derived cells express CAM 5.2 staining at cytoplasm level (98 ± 6% positive cells). As expected, no CD140 labelling was found in any of the samples due to the absence of contaminating fibroblasts.

**Figure 4:**
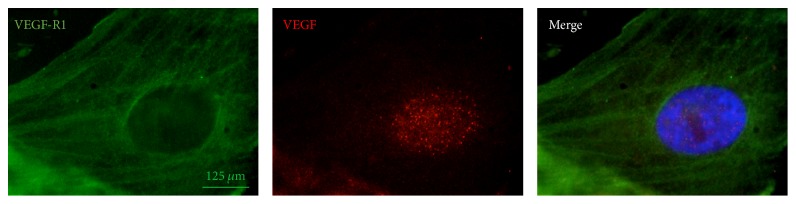
Immunofluorescence analysis of cultured human keratinocytes derived from pterygium explants labelled with anti-VEGF (red label at nuclear level 78 ± 3%) and anti-VEGF-R1 (green label located both in the nucleus and cytoplasm 96 ± 7%). Original magnification: 100x. The observations were carried out using a ZEISS Axioskop light microscope equipped with a Coolsnap video camera to acquire images analysed with the MetaMorph 6.1 Software (Universal Imaging Corp, Downingtown, PA). Images displayed are representative of 3 independent experiments.

To investigate the presence of angiogenic pathways in pterygium-derived epithelial cells that could result in neovascularization and pterygium development and growth, immunofluorescence staining for VEGF and VEGF-R1 was performed.  Figure 4 shows the presence of VEGF-R1 (green label) in both the nucleus and the cytoplasm (96 ± 7% positive cells) whereas VEGF (red label) is present only in the nuclear compartment (78 ± 3% positive cells).

### 3.4. Effects of* Curcuma longa* on Pterygium-Derived Keratinocytes

To assess the effects induced by* Curcuma longa* administration, samples were treated with 1.3%* Curcuma longa* dissolved in 0.001% BCA up to 24 h and compared with samples treated with BCA alone or left untreated as controls.

**Figure 5:**
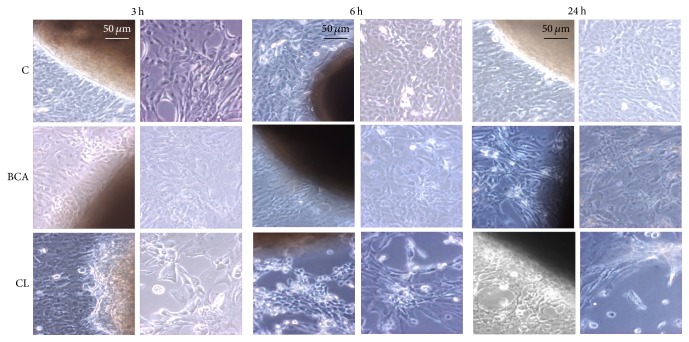
Effect of BCA and* Curcuma longa* (CL) on human keratinocytes derived from pterygium explants after 3, 6, and 24 h treatment. Loss of adhesion and morphological changes occur in a time-dependent manner in treated samples. Original magnification: 20x. Observations were carried out with a phase contrast light microscope (LEICA) equipped with a CoolSNAP video camera to acquire computerized images (Photometrics). The brown/dark regions are pterygium explants in culture. Images displayed are representative of 5 independent experiments.

As shown by phase contrast microscopy (Figure 5), clear changes in cell morphology occurred as early as after 3 h treatment with* Curcuma longa* leading a number of cells to lose the cobblestone-like appearance and to detach from the culture dish, resembling the typical hallmarks of apoptotic cell death. Interestingly, detachment started at the periphery of the culture dish moving towards the explant in a time-dependent manner. On the other hand, samples treated with BCA alone displayed the presence of granular material and cell debris due to the occurrence of necrotic cell death.

**Figure 6:**
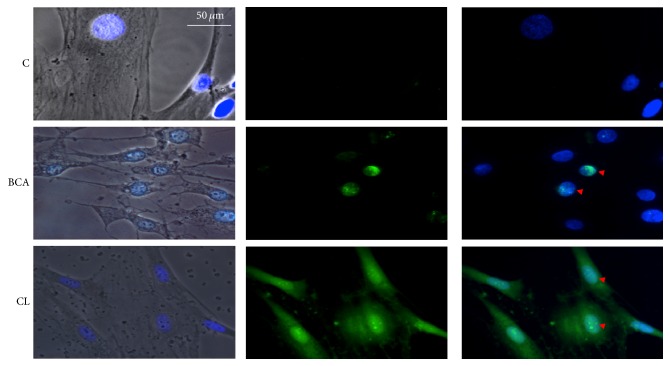
Immune fluorescence labelling of human keratinocytes derived from pterygium explants after treatment with BCA and* Curcuma longa* (CL) for 24 h. A significantly increased positivity for the terminal deoxynucleotidyl transferase- (TdT-) mediated nick end labelling (TUNEL) was found in samples treated with BCA (27 ± 2%). In* Curcuma longa*-treated samples a different pattern of apoptosis was revealed due to the presence of DNA also in the cytoplasm (93 ± 7%). Red arrow heads indicate some apoptotic cells. Nuclei were counterstained with 6-diamino-2-phenylindole (DAPI) (blue fluorescence). Green and blue fluorescence single emissions are overlapped in the merge panels. Original magnification: 40x. Images displayed are representative of 3 independent experiments.

To better evaluate the possible apoptotic effects induced by the different treatments (*Curcuma longa* plus BCA or BCA alone), samples were subjected to TUNEL assay. Analysis of DNA strand breaks revealed the absence of apoptosis induction in untreated control cells (1 ± 0.2%); conversely BCA treatment showed a slight increase in TUNEL^pos^ cells (27 ± 2%, *p* < 0.05 versus Control) whereas* Curcuma longa*-treated samples showed a significantly increased number of TUNEL^pos^ cells (93 ± 7%; *p* < 0.03 versus Control; *p* < 0.05 vs BCA). Of note, in* Curcuma longa*-treated samples the fluorescent labelling was both located at nuclear level and finely dispersed in the cytoplasm of apoptotic cells (Figure 6).

**Figure 7:**
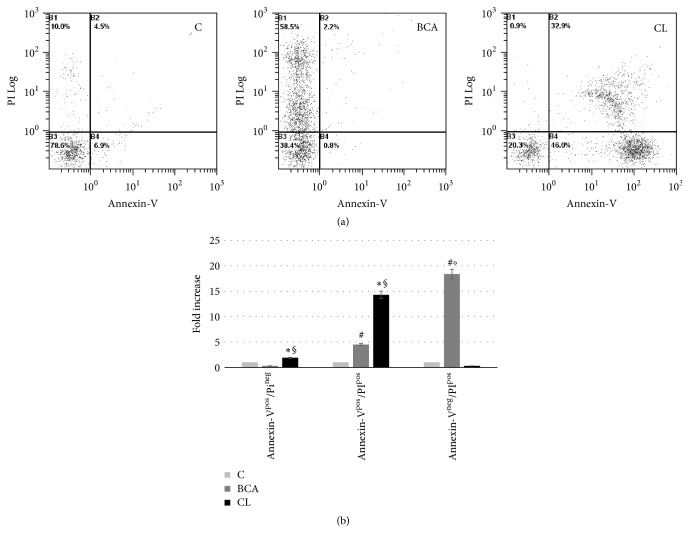
Annexin-V/PI evaluation in human keratinocytes derived from pterygium explants. (a) Flow cytometric dot plots of one representative experiment. Apoptotic cells can be discriminated from viable, late apoptotic or necrotic cells according to their fluorescence emission. B3: viable cells; B4: early apoptotic cells; B2: late apoptotic cells; B1: necrotic cells. (b) The histogram shows the fold increase of Annexin-V^pos^/PI^neg^, Annexin-V^pos^/PI^pos^, and Annexin-V^neg^/PI^pos^ cells ± SD of three independent experiments (^#^*p* < 0.0214 BCA versus C; ^*∗*^*p* < 0.0497 CL versus C; ^§^*p* < 0.0459 CL versus BCA; °*p* < 0.0288 BCA versus CL).

Finally, flow cytometric analysis of Annexin-V/PI staining was performed to further confirm the occurrence of apoptotic and necrotic cell death after* Curcuma longa* and BCA treatment of pterygium-derived keratinocytes. In apoptotic cells, the membrane phospholipid phosphatidylserine (PS) is translocated from the inner to the outer leaflet of the plasma membrane. Annexin-V is a Ca^2+^-dependent phospholipid binding protein with high affinity for PS that is exposed on the cell surface of apoptotic cells. PS membrane translocation precedes the loss of membrane integrity, which accompanies the later stages of cell death resulting from either apoptotic or necrotic processes. Annexin-V is typically used in combination with PI, which can enter the nucleus due to cell membrane permeability changes occurring in the later stage of apoptosis or necrosis. This analysis revealed that the percentage of viable cells decreased in the presence of BCA as well as of* Curcuma longa*. As shown in Figures 7(a) and 7(b) the 24 h treatment with* Curcuma longa* lead to an increase in early (Annexin-V^pos^/PI^neg^) and late (Annexin-V^pos^/PI^pos^) apoptotic cells compared to untreated controls (for both *p* < 0.05) and BCA-treated samples (for both *p* < 0.05), whereas the number of necrotic cells (Annexin-V^neg^/PI^pos^ cells) was significantly higher in samples treated with BCA alone compared to controls (*p* < 0.03) and to* Curcuma longa* (*p* < 0.05). In fact, after* Curcuma longa* treatment, the percentage of Annexin-V^pos^/I^pos^ cells was 14.3 fold higher than control (*p* < 0.05) whereas after BCA treatment the percentage of Annexin-V^pos^/PI^pos^ cells was 4.5-fold higher than untreated controls (*p* < 0.03) and, more importantly, the percentage of Annexin-V^neg^/PI^pos^ cells was even 18.4-fold higher compared to untreated controls (*p* < 0.03).

## 4. Discussion

Pterygium is a common ocular surface disease, which is believed to be closely associated with ultraviolet exposure [22]. Until recently, surgery was considered as the only feasible option of pterygium treatment. However, apart from being invasive and associated with a variety of potential serious complications, surgery often results in a more aggressive clinical behaviour of pterygium [9]. Here we propose an alternative strategy for the removal of human pterygium based on the dramatic apoptogenic effect of* Curcuma longa* on human pterygium-derived keratinocytes. In fact, as early as after 3 h of* in vitro* treatment pterygium-derived keratinocytes incubated even with a low concentration (1.3%) of the total extract of* Curcuma longa* are able to undergo apoptotic cell death as shown with light microscopy, TUNEL assay, and Annexin-V/PI staining in flow cytometry. Instead, BCA, used as a single treatment, induces keratinocytes to necrotic cell death that, unlike apoptosis, is a type of inflammatory cell death. Cells undergoing apoptosis were recognizable in light microscopy for their typical shrinkage leading them to detach very early from the plastic surface of the culture dish. In addition, typical hallmarks of apoptotic cell death, including DNA strand breaks and phosphatidylserine exposition at the cell membrane, were detected with the TUNEL technique and with Annexin-V/PI staining, respectively. Interestingly, the use of* Curcuma longa* in combination with BCA was able to attenuate necrotic effects of BCA used as a single treatment. We cannot assign this apoptogenic effect to specific components of* Curcuma longa* since it is known that this compound contains a number of curcuminoids including mainly curcumin. Curcumin is considered the active principle of* Curcuma longa* due to its well-recognized anti-inflammatory effects [17]. In fact, studies carried out by other laboratories have identified a number of different molecules involved in inflammation that are inhibited by curcumin including phospholipase, lipoxygenase, cyclooxygenase 2, leukotrienes, thromboxane, prostaglandins, nitric oxide, collagenase, elastase, hyaluronidase, monocyte chemoattractant protein-1 (MCP-1), interferon-inducible protein, tumour necrosis factor (TNF), and interleukin-12 (IL-12) [23]. More recently, it has become evident that curcumin also modulates survival-associated signalling pathways causing apoptosis [17, 19] and exerting potent anticancer effects [24]. Moreover, curcumin has been demonstrated in six human trials to be highly safe when orally administered [22] and to significantly inhibit the proliferation of human pterygium fibroblasts, arresting them in G_0_/G_1_ phase and inducing apoptosis in a dose- and time-dependent manner [25]. Our results together with the findings of Zhang et al. [25] pave the way to a possible medical treatment of pterygium and are particularly interesting in light of previous studies showing that rates of proliferation in pterygium samples are similar to those found in the normal conjunctival surface, suggesting that pterygium may represent a failure of appropriate apoptosis [26]. Furthermore, our study confirms previous observations highlighting the expression, in pterygium keratinocytes, of nuclear VEGF [27–29] and gives evidence for the first time to the expression of nuclear and cytoplasmic VEGF-R1. These observations, although still to be further characterized, are particularly intriguing since they suggest a possible role of epithelial cells in new blood vessels formation that is one of pathogenetic mechanisms involved in pterygium growth and recurrence [26]. In fact, as a benign type of uncontrolled cell proliferation, pterygium requires cell migration and local angiogenesis that is triggered and controlled by several angiogenic and inhibitory factors [8]. Among these factors, VEGF is a potent angiogenic cytokine that has a pivotal role in normal and pathological angiogenesis [30] by interacting with three different receptors VEGF-R1, VEGF-R2, and VEGF-R3 [1]. VEGF-R2 appears to mediate almost all of the known cellular responses to VEGF, whereas the function of VEGF-R1 is less well defined, although it is thought to modulate VEGF-R2 signalling, acting as a dummy/decoy receptor, and to promote angiogenesis mainly by increasing bone marrow-derived macrophage recruitment [31]. In this respect, it is worth noting that curcumin appears to be able to inhibit tumour growth and vasculogenesis* in vivo* through interrupting VEGF/VEGF-R2 signalling pathway [32].

All in all, previous and present findings suggest that* Curcuma longa* or its components could have a promising therapeutic potential in the pterygium treatment by interacting both with apoptotic disorder and, potentially, with blood vessels formation. Further studies are needed to better clarify the pharmacokinetics and tolerability in the human eye of this nutraceutical compound.
